# Effects of norepinephrine infusion on cerebral energy metabolism during experimental haemorrhagic shock

**DOI:** 10.1186/s40635-022-00432-z

**Published:** 2022-02-04

**Authors:** Rasmus Peter Jakobsen, Elisabeth Charlotte Hansen, Troels Halfeld Nielsen, Carl-Henrik Nordström, Palle Toft

**Affiliations:** 1 Department of Anaesthesiology and Intensive Care, Odense University Hospital, J.B. Winsløws Vej 4, indgang 8, indgang 5, Penthouse/2, 20, 201, 5000 Odense C, Denmark; 2 Faculty of Health Sciences, University of Southern Denmark, J.B. Winsløws Vej 19 3, 5000 Odense C, Denmark; 3 Department of Neurosurgery, Odense University Hospital, Kløvervænget 47, indgang 44, 1. etage, 5000 Odense C, Denmark; 4 Faculty of Medicine, Lund University, P.O. Box 117, 221 00 Lund, Sweden

**Keywords:** Haemorrhagic shock, Norepinephrine, Microdialysis, Cerebral metabolism, Trauma

## Abstract

**Background:**

The use of norepinephrine in the case of life-threatening haemorrhagic shock is well established but widely discussed. The present study was designed to compare the effects of early norepinephrine treatment vs. no treatment on cerebral energy metabolism during haemorrhagic shock.

**Methods:**

Twelve pigs were subjected to haemorrhagic shock, 4 in the control group and 8 in the norepinephrine (NE) group. Following a 60 min baseline period haemorrhagic shock was achieved by bleeding all animals to a pre-defined mean arterial blood pressure (MAP) of approximately 40 mm Hg. When mean arterial pressure had decreased to 40 mmHg NE infusion started in the treatment group. After 90 min, NE infusion stopped, and all pigs were resuscitated with autologous blood and observed for 2.5 h. During the experiment cerebral tissue oxygenation (PbtO_2_) was monitored continuously and variables reflecting cerebral energy metabolism (glucose, lactate, pyruvate, glutamate, glycerol) were measured by utilizing intracerebral microdialysis.

**Results:**

All 12 pigs completed the protocol. NE infusion resulted in significantly higher MAP (*p* < 0.001). During the shock period lactate/pyruvate (LP) ratio group increased from 20 (15–29) to 66 (38–82) (median (IQR)) in the control group but remained within normal limits in the NE group. The significant increase in LP ratio in the control group remained after resuscitation. After induction of shock PbtO_2_ decreased markedly in the control group and was significantly lower than in the NE group during the resuscitation phase.

**Conclusion:**

NE infusion during haemorrhagic shock improved cerebral energy metabolism compared with no treatment.

## Background

The prevailing regimen in treating patients in haemorrhagic shock is damage control resuscitation and damage control surgery [1–5]. Damage control resuscitation incorporates different strategies as minimized use of crystalloids, tranexamic acid, balanced blood-transfusion, and permissive hypotension [6]. During permissive hypotension the clinician allows a lower target-mean arterial pressure (MAP), to avoid adverse effects, such as dilutional coagulopathy or acceleration of haemorrhage [7–9]. A decrease in mean arterial pressure (MAP) below the lower autoregulatory limit will result in a decrease in the cerebral perfusion pressure, which could compromise cerebral energy metabolism [10]. However, the use of vasopressors in haemorrhagic shock is still controversial [11]. Some guidelines promote the use of vasoactive drugs in the face of life-threatening haemorrhage, whereas others warn against them [12–14].

The objective of this study was to investigate the effect of the vasopressor norepinephrine on MAP, cerebral energy metabolism evaluated from intracerebral microdialysis, cerebral tissue oxygen tension (PbtO_2_) and intracranial pressure (ICP) in an experimental model of induced haemorrhagic shock. The experimental model has been used in previous studies of cerebral energy metabolism during haemorrhagic shock [15, 16].

## Materials and methods

The study was designed as a non-randomised, non-blinded experimental study using pigs 4 months of age weighing approximately 40 kg. Twelve female Danish Landrace mix pigs were allowed to acclimatise for 1 week at the biomedical laboratory. At the day of the experiment, they had fasted overnight with free access to water. Four pigs were allocated to the control group, and 8 pigs in the group treated with the vasopressor noradrenalin (NE). Approval of the study had been given from The Danish Animal Experiments Inspectorate (2020-15-0201-00478).

### Anaesthesia, mechanical ventilation, and surgical preparation

The porcine model of haemorrhagic shock and the surgical preparations have been described previously [16–18]. Instrumentation and anaesthesia were the same in the control group and NE group. Sedation was achieved with an intramuscular injection of midazolam (0.25 mg/Kg) ketamine (5 mg/Kg) medetomidine (0.03 mg/Kg), butorphanol (0.1 mg/Kg) and atropine (0.025 mg/kg). Standard 18G intravenous cannulas were placed in veins in both ears. Anaesthesia was induced with intravenous midazolam (0.625 mg/Kg) and ketamine (12.5 mg/Kg) and maintained with an infusion of midazolam (5 mg/Kg/h) and fentanyl (50 µg/Kg/h).

Arterial blood gas samples were analysed every 30 min for pH, PaO_2_, PaCO_2_, lactate, Ca^2+^ and haemoglobin (EPOC blood analyser, Woodley Equipment Company Ltd, England).

### Cerebral monitoring

A microdialysis catheter (CMA 70 Bolt MDialysis, Stockholm, Sweden) was inserted through a small burr hole in the left hemisphere and perfused with artificial CSF (M Dialysis AB, Stockholm, Sweden) at a rate of 0.3 μL/min (CMA 106 MD pump, MDialysis AB, Stockholm, Sweden). The dialysates were collected in microvials and immediately analysed for glucose, lactate, pyruvate, glutamate, and glycerol every 30 min using an Iscus Flex analyser (M Dialysis AB, Stockholm, Sweden).

To monitor brain tissue oxygenation (PbtO_2_), a probe (Licox CC1SB, Integra Neurosciences Ltd. NJ, USA) was introduced to the right hemisphere through a small burr hole. PbtO_2_ data were collected using the AC3.1 monitor (Integra Neurosciences Ltd.).

A transducer for monitoring intracranial pressure (ICP) was placed in the left hemisphere (Integra Neurosciences Ltd. New Jersey, USA) ICP was monitored continuously, and data were collected by a CAM01 monitor (Integra Neurosciences Ltd. New Jersey, USA).

### Experimental protocol

**Fig. 1:**
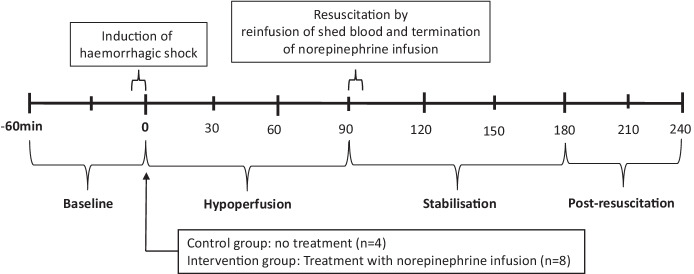
Experimental protocol timeline. Note that the experimental protocol is divided into four intervals. Interval 1: Baseline − 60–0 min. Interval 2: Hypoperfusion period 0–90 min, Interval 3: Stabilisation period 90–180 min in which physiological state might return to homeostasis. Interval 4: post-resuscitation period 180–240 min

The experimental protocol is illustrated in Fig. 1. After preparation and insertion of all probes, the animals were allowed a minimum of 1 h to stabilize before start of the hypoperfusion period. Vital parameters were recorded every 10 min. Following a 60 min baseline period haemorrhagic shock was achieved by bleeding the animals to a pre-defined MAP of approximately 40 mm Hg at a rate of 2.15 mL/kg/min over 7 min, and then 1.15 mL/kg/min over the remaining period [15, 16]. The depth and duration of haemorrhagic shock necessary for producing cerebral ischemia that caused compromised energy state was based on previous experiments [15]. At the end of the experimental protocol the animals were euthanatized under anaesthesia with pentobarbital 200 mg/mL in concentrated ethanol.

The control group experiments were the first to be conducted. Animals in the control group were kept at a MAP of about 40 mmHg by withdrawing or infusing After induction of shock and MAP = 40 mmHg was reached, we continued to withdraw or reinfuse shed blood to keep a MAP of 40 mmHg. The shed blood was stored in a citrated glucose solution at 37 °C. The amount of blood withdrawn or reinfused which was needed to maintain a MAP = 40 mmHg were recorded in intervals of 10 min. Following 90 min of haemorrhagic shock, the animals were resuscitated by re-infusing the remaining shed blood at a rate of 120 mL/min until all blood was returned. After resuscitation the pigs were observed for 150 min (Fig. 1). When animals in the NE group after haemorrhage had reached a MAP of 40 mmHg a weight adjusted infusion with norepinephrine (0.03 mg/kg in 50 ml isotonic NaCl) was started. The rate of norepinephrine infusion was titrated until a MAP of 80 mmHg was reached. During the following 90 min blood was withdrawn or reinfused in the same amount and rate as determined during the control group experiments. Following the 90 min of haemorrhagic shock in the control group, the animals were resuscitated by re-infusing the remaining shed blood at a rate of 120 mL/min until all blood was returned. After resuscitation norepinephrine was titrated down and finally terminated (Fig. 1).

## Statistics

The time course of the experimental protocol was divided into four separate periods (Fig. 1). The discrimination between the hypoperfusion and resuscitation period is the time when all the shed blood has been reinfused. The post-resuscitation period was arbitrary defined as the last 60 min of the experimental protocol. Experiences from earlier studies have showed that there is a need for a stabilisation period after a period with hypoperfusion, in which the cerebral metabolism might return to normal. Data were modelled with a mixed effect model for repetitive measurements using treatment with NE as a fixed effect, and each animal as a random effect. Non-repetitive data were tested with a Mann Whitney U test. Unless otherwise noted data is reported as median and interquartile range (25–75 percentiles). We did not calculate sample size due to the explorative nature of this study. All *p* values are to be considered exploratory, and therefore, no adjustment for generating multiple *p* values was made. A *p* value less than 0.05 is considered statistically significant. The data analysis and graphs were carried out in STATA 16 statistical software. (StataCorp, College station, Texas USA).

## Results

All 12 pigs completed the experimental protocol. The weight of the pigs in the control group and NE group was 43.5 kg (40.25–46.65 kg) and 41.9 kg (40.3–44.5 kg), respectively (*p* = 0.386). The total amount of drained blood in the control group was 26.55 mL/Kg (23.2–32.9 ml/Kg) and in the NE group 21.36 ml/Kg (18.4–25.9 ml/Kg) (*p* = 0.1649). The drained volumes corresponded to between 33 and 41% of the total blood volume [21]. The total dose of norepinephrine in the NE-group was 0.528 mg (0.475–2.595 mg).

### Systemic physiological and biochemical variables

**Fig. 2:**
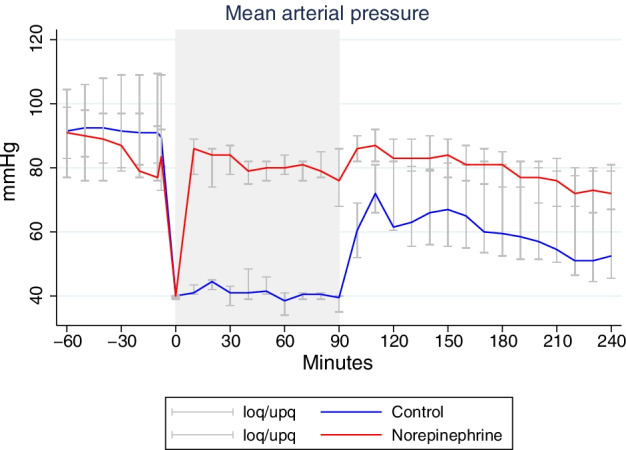
Dynamic changes of median (interquartile range) mean arterial pressure (MAP) throughout the experiment. NE group *n* = 8. Control group *n* = 4. Note the steep decrease in MAP in both groups at the start of hypoperfusion period. In addition, note the normalisation of MAP after start of NE infusion. Shaded area illustrates hypoperfusion period

Table 1 shows systemic physiological and biochemical variables as well as PbtO_2_ for both experimental groups during baseline, hypoperfusion and post resuscitation. During baseline all variables were within normal ranges in both groups. After initiation of bleeding, MAP decreased to 40 mmHg in both groups (Fig. 2). Treatment with NE caused a rapid increase in MAP in the NE group which remained during the hypoperfusion period (Table 1). After reinfusion of shed blood, a rapid increase in MAP was observed in the control group. However, a significant difference in MAP persisted after resuscitation and termination of NE infusion (Table 1). The time course for the changes in MAP in both groups are shown in Fig. 2.

**Table 1:** General physiological haemodynamic and systemic parameters for baseline, hypoperfusion and post resuscitation periods

Interval	Control	NE	Control vs. NE group *p* value
Baseline (− 60)–0 min			
MAP (mmHg)	88 (78–98)	78 (72–95)	0.198
HR (bpm)	56 (55–87)	87 (74–103)	0.016*
PbtO2 (mmHg)	22 (8–70)	37 (30–40)	0.880
PaO2 (kPa)	53 (49–57)	37 (36–40)	0.001*
PaCO2 (kPa)	5.6 (5.4–6)	6.4 (5.9–6.7)	< 0.001*
b-Hemoglobin (mM/L)	5.8 (5.6–6.3)	5 (4.8–5.3)	< 0.001*
b-Glucose (mM/L)	4.1 (3.7–6)	5.8 (3.9–8.1)	0.180
b-Lactate (mM/L) ABG	0.9 (0.5–1)	0.5 (0.4–0.9)	0.753
b-pH ABG	7.45 (7.44–7.48)	7.46 (7.43–7.5)	0.951
Diuresis (mL)	25 (0–72.5)	0 (0–50)	0.274
Hypoperfusion 0–90 min			
MAP (mmHg)	41 (40–42)	80 (75–86)	< 0.001*
HR (bpm)	108 (63–180)	98 (85–115)	0.625
PbtO2 (mmHg)	8 (4–18)	28 (23–34)	0.160
PaO2 (kPa)	52 (46.6–53.9)	38 (35–41)	0.019
PaCO2 (kPa)	5.5 (5.2–6.3)	6.3 (6.1–6.7)	0.031*
b-Hemoglobin (mM/L)	5.7 (5–6.1)	5.1 (4.8–5.6)	0.189
b-Glucose (mM/L) MD	6 (3.8–7.0)	4.9 (4.2–7.0)	0.974
b-Lactate (mM/L) ABG	8.2 (2.7–13.7)	1.1 (0.9–1.7)	0.001*
b-pH ABG	7.39 (7.23–7.4)	7.46 (7.43–7.49)	0.013
Diuresis (mL)	0 (0.35)	11.5 (0–47.5)	0.380
Post resuscitation 180–240 min			
MAP (mmHg)	56 (47–79)	74 (69–80)	0.037
HR (bpm)	89 (76–107)	90 (76–99)	0.858
PbtO2 (mmHg)	7 (2–10)	29 (13–34)	0.021
PaO2 (kPa)	51(49–54)	38 (37–42)	< 0.001*
PaCO2 (kPa)	6.5 (5.3–6.8)	6 (5.7–6.5)	0.665
b-Hemoglobin (mM/L)	6.5 (6–6.7)	4.6 (4.2–4.9)	< 0.001*
b-Glucose (mM/L)	2.8 (0.7–3.2)	3.6 (2.9–4.3)	0.432
b-Lactate (mM/L) ABG	3.9 (2–8.4)	0.5 (0.3–0.6)	0.003*
b-pH ABG	7.32 (7.29–7.39)	7.47 (7.45–7.51)	< 0.001*
Diuresis (mL)	0 (0–30)	0 (0–4)	0.676

NE group *n* = 8, control group *n* = 4. Values are medians (interquartile range). Test statistics performed with mixed effect model

*Statistical significant* p* value < 0.05

### *Intracerebral variables: Cerebral energy metabolism and PbtO*_*2*_

Table 2 shows median (IQR) levels for all intracerebral biochemical variables. During baseline there was no significant difference between control and NE group. During the period of hypoperfusion significant increases in lactate, glutamate, glycerol and LP ratio were obtained simultaneously with a significant decrease in glucose. These significant differences remained during the post resuscitation period.

**Table 2:** Biochemical variables obtained on microdialysis from cerebral hemisphere

Baseline	Location	Control	NE	Control vs. NE group *p* value
LP ratio	Hemisph	20 (15–29)	23 (18–27)	0.834
Lactate (mM/L)	Hemisph	2.2 (1.4–3.8)	2.1 (1.7–3.1)	0.670
Pyruvate (mM/L)	Hemisph	100 (92–134)	92 (73–108)	0.643
Glucose (mM/L)	Hemisph	2.5 (1.8–2.8)	2.6 (2.2–3.2)	0.183
Glutamate (µM/L)	Hemisph	15 (9–72)	12 (4–20)	0.081
Glycerol (µM/L)	Hemisph	93 (58–149)	62 (45–90)	0.106

Hypoperfusion	Location	Control	NE	Control vs. NE group *p* value
LP ratio	Hemisph	66 (38–82)	24 (19–31)	< 0.001*
Lactate (mM/L)	Hemisph	7.8 (6.2–12.3)	2.5 (2.1–3.6)	< 0.001*
Pyruvate (mM/L)	Hemisph	104 (81–177)	112 (91–132)	0.670
Glucose (mM/L)	Hemisph	1 (0.4–1.6)	2.9 (2.4–3.9)	< 0.001*
Glutamate (µM/L)	Hemisph	93 (6–155)	8 (4–11)	0.001*
Glycerol (µM/L)	Hemisph	190 (75–270)	59 (44–86)	< 0.001*

Post resuscitation	Location	Control	NE	Control vs. NE group *p* value
LP ratio	Hemisph	73 (29–100)	25 (21–29)	0.013*
Lactate (mM/L)	Hemisph	4.9 (1–6.5)	1.3 (0.8–1.6)	< 0.001*
Pyruvate (mM/L)	Hemisph	102 (71–185)	108 (100–133)	0.972
Glucose (mM/L)	Hemisph	0.7 (0.5–0.8)	2.3 (1.6–2.9)	0.003*
Glutamate (µM/L)	Hemisph	47 (6–110)	4 (2–7)	0.005*
Glycerol (µM/L)	Hemisph	461 (254–545)	42 (23–55)	< 0.001*

Values are divided into three-time intervals and expressed as median (interquartile range). Control *n* = 4. NE group *n* = 8. Test statistics performed with mixed effectt model

*Statistical significant* p* value < 0.05

**Fig. 3:**
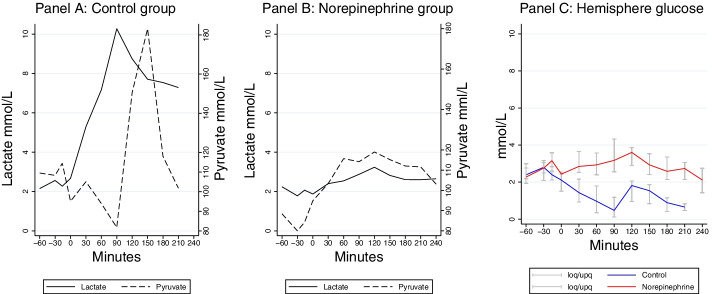
Median (interquartile range) values of lactate, pyruvate and glucose concentrations in the hemisphere in the controls and NE group during the experiment. Note that the increase in LP ratio in the control group during hypoperfusion is due to an increase in lactate simultaneously with a decrease in pyruvate. After resuscitation the lactate levels in the control group remains elevated but he pyruvate levels trends towards normalising. In addition, note the normalisation of glucose in the control group after resuscitation. Control *n* = 4. NE group *n* = 8; *loq* lower quartile, *upq* upper quartile

The time courses for the changes in lactate, pyruvate and glucose during the study are illustrated in Fig. 3. During the hypoperfusion period (0–90 min) lactate increased dramatically in the control group simultaneously with a decrease in pyruvate. During the initial phase of resuscitation (90–180 min) lactate remained high with a profound increase in pyruvate. This was followed by a second decrease in pyruvate, while lactate remained elevated. In the NE group a slight increase in lactate and a moderate increase in pyruvate was observed during these phases (0–240 min). During hypoperfusion and resuscitation glucose decreased in the control group but remained essentially unchanged in the NE group.

During hypoperfusion and post resuscitation the levels of glutamate and glycerol increased markedly in the control group (Table 2). In the NE group these variables remained virtually unchanged.

During the baseline period median PbtO_2_ was lower in the control than in the NE group (22 vs. 37 mmHg) but the difference did not reach statistical significance (Table 1). The decrease in median PbtO_2_ was in the control group more pronounced during hypoperfusion (8 vs. 28 mmHg) and the difference was even more explicit and statistically significant in the post resuscitation period (7 vs. 29 mmHg) (Table 1). During the latter part of the hypotension period (40–90 min) a marked decrease in PbtO_2_ was observed in the control group. Immediately after resuscitation a transitory increase was obtained (100–120 min) followed by a secondary marked decrease (130–240 min). In the NE group a moderate, stabile decrease in PbtO_2_ occurred during the whole study (0–240 min).

During the experiment ICP was recorded every 10 min. In the NE group all data were recovered: baseline 3 mmHg (− 2–8 mmHg), hypoperfusion 6 mmHg (2–8 mmHg) and post resuscitation 12.5 mmHg (7–17 mmHg). Due to equipment failure ICP data from the control group were lost. Accordingly, a comparison of the two groups was impossible.

## Discussion

The use of vasopressors in haemorrhagic shock is controversial [11–14]. Widely different experiences regarding the effects of noradrenaline on cerebral blood flow (CBF) and cerebral energy metabolism have been reported [21–24]. In the present study we explore the effects on biochemical variables related to cerebral energy metabolism when noradrenaline is used to restore MAP acutely during haemorrhagic shock for 90 min before reinfusion of the shed blood to restore the circulating volume (Fig. 1).

Measurements of PbtO_2_ represent the product of CBF and the cerebral arterio-venous difference in oxygen tension rather than a direct measurement of total oxygen delivery or cerebral oxygen metabolism [25]. In the present experimental situation this implies that PbtO_2_ may be regarded as a qualitative reflection of CBF. Under normal conditions the numerical value of PbtO_2_ obtained varies and a definite lower acceptable limit is not possible to define: the limit for “cerebral ischemia” has in various publications been defined at levels from 5 to 23 mmHg and normal values between 15 and 42 mmHg have been reported in experimental studies [26]. In the present study median PbtO_2_ was slightly lower in the control group (Table 1). As cytoplasmatic redox state evaluated by LP ratio was similar in the experimental groups tissue oxygenation was in both groups sufficient for normal energy metabolism (Table 2). Accordingly, we regard the initial non-significant difference in PbtO_2_ as irrelevant for the interpretation of observed changes in biochemical variables.

In the control group PbtO_2_ decreased to below 10 mmHg during the latter part of the hypoperfusion period indicating a very pronounced decrease in CBF. After resuscitation a transient increase in PbtO_2_ indicated a temporary increase in blood flow followed by a final period of very low CBF. In the NE group PbtO_2_ exhibited a moderate, stable decrease in PbtO_2_ indicating relatively well preserved CBF. Our data regarding the effect of NE on PbtO_2_ are in agreement with the study by Meybohm et al*.* in piglets [27]. However, these authors concluded that although NE increased PbtO_2_ the improvement was probably not sufficient to restore cerebral energy metabolism. In the present study we can relate PbtO_2_ to cerebral biochemical variables reflecting energy metabolism.

Cytoplasmatic red-ox state is described by the lactate/pyruvate ratio (LP ratio) [28]. In the present study LP ratio was almost identical in the two study groups during baseline (Table 2). About 30 min after start of the hypoperfusion period the LP ratio increased rapidly in the control group due to a marked increase in lactate simultaneously with a decrease in pyruvate (Fig. 3). A delay in the detection of biochemical changes is expected as the microdialysis perfusion fluid must be transported from the dialysis catheter to the collecting vial before the chemical analyses can be performed. However, the increase in LP ratio coincided with a pronounced decrease in PbtO_2_, indicating a dramatic reduction in CBF. Accordingly, the delayed biochemical deterioration observed in the control group is in this study not a technical artifact caused by the microdialysis technique.

The biochemical and physiological variables document that in the control group the hypoperfusion period caused profound cerebral ischemia which was avoided in the NE group. The increase in glutamate in the control group indicates that cerebral energy metabolism was insufficient to cover the tissue demands and the increase in glycerol shows that irreversible degradation of cellular membranes had occurred [29–31]. In the NE group biochemical variables remained unchanged demonstrating that during induced haemorrhagic shock acute infusion of NE will not only increase MAP but also preserve PbtO_2_ (CBF) and prevent biochemical signs of cerebral ischemia, compromised oxidative energy metabolism and signs of cellular degradation.

The results of the present study may be compared with similar experimental studies. In their study Meybohm et al. [27] observed that NE increased PbtO_2_ but the effect on cerebral energy metabolism was not directly investigated. When comparing the effects of either epinephrine or vasopressin during induced hypotension and resuscitation Küchler et al. [32] observed no differences between the two treatments regarding restoration of cerebral hemodynamic and energy metabolism after induced haemorrhagic shock. However, in their study the level of hypotension was not sufficient to cause biochemical signs of ischemia. The beneficial effects of NE in the present study are interpreted as caused by the increase in MAP and CBF. A direct effect of NE cerebral energy metabolism is unlikely. It has been demonstrated that the direct effect of the catecholamines adrenaline and noradrenaline result in an increase in CBF and cerebral metabolic rate at virtually unchanged levels in biochemical variables related to cerebral energy metabolism [21, 33].

The beneficial effects of NE may be of clinical importance also in other conditions with a dangerous decrease in cerebral perfusion pressure. It has been shown that following cardiac standstill and resuscitation many patients exhibit compromised cerebral energy metabolism due to ischemia/mitochondrial dysfunction up to 20 h after return of spontaneous circulation [34]. The data indicate that initial brain recirculation is often insufficient due to too low MAP/cerebral perfusion pressure. Conflicting results regarding the possible beneficial effect of vasopressor therapy in after cardiac arrest have recently been published. In a clinical study it was shown that incremental doses of norepinephrine increased blood pressure and systemic vascular resistance without affecting cardiac output or cerebral oxygenation [35]. However, an experimental study of cardiac arrest reported that adrenaline administration increased cerebral perfusion pressure and regional CBF as well as cerebral oxygenation and energy metabolism [36].

## Limitations

The PbtO_2_ and MD probes in the hemisphere will always provide a local estimate of ischemia in the given area and may over/underestimate global conditions. To achieve equivalent measurements across all animals, it was striven to methodically place all probes at the same location in all experimental animals. In the clinical setting, it is standard to verify correct positioning in white matter by CT scans. Unfortunately, this was not possible in our laboratory. Second, we assume conditions to be equally distributed across the cerebrum and would only expect small regional differences in PbtO_2_ and microdialysis measurements. If we had continued to monitor the animals for a longer period it is possible that some of the parameters would have normalized. However, even though a biochemical normalization would have occurred, it does not eliminate the fact that the animal had been subjected to an ischemic event which might lead to focal or cognitive deficits. MAP 40 mmHg for 90 min would probably not be accepted in a clinical situation without interventions. The experimental model is thus only limited transferable in a clinical context as we focused on treatment with norepinephrine alone without any other interventions, such as fluids or blood transfusions. The authors chose the length of hypotension, because earlier studies had showed that 90 min hypotension with a MAP of 40 mmHg resulted in an irreversible metabolic crisis. In this context we would be able to observe if norepinephrine would worsen or better cerebral metabolic state. One drawback of the present study is that MAP of the NE group exceeded what would normally be targeted during haemorrhagic resuscitation. Treatment with norepinephrine resulted in a MAP that was comparable with measurements made at baseline. When using norepinephrine in a clinical situation, the target MAP would often be the same as in permissive hypotension, i.e., MAP of 50–60 mmHg. However, one of the concerns of using vasopressors during haemorrhagic shock is the risk of cerebral vasoconstriction and reduced CBF. (31–37) Most likely, the preserved cerebral metabolism is a result of increased MAP and thus increased CPP. However, if norepinephrine causes cerebral vasoconstriction, the larger doses needed for obtaining a higher MAP would probably have resulted in a decrease in CBF and eventually in compromised delivery of oxygen and glucose to the brain, which would have been detected by microdialysis.

## Strengths

We chose a combination of fixed pressure and fixed volume model for haemorrhagic shock. The fixed volume model does not consider intraindividual variations in total blood volume or intraindividual adaptations to blood loss. The total amount of blood withdrawn at induction of shock ranged from 701 to 1654 ml indicating that different amounts of blood withdrawn was needed to reach the same physiological endpoint of MAP 40 mmHg. A fixed pressure model relies on physiological parameters, rather than a fixed volume. In a clinical setting it is rare to address the level of haemorrhagic shock by quantifying volume of blood lost, instead clinicians evaluate severity of shock by the physiological response. However, since it was not possible to maintain a “true” MAP during treatment with norepinephrine we used a fixed volume determined by the control group during this phase of the experiment.

## Conclusion

Early treatment with NE during severe and prolonged haemorrhagic shock restored MAP and chemical variables related to cerebral energy metabolism. As evaluated from the biochemical variables, treatment with NE in hypovolemic shock did in this experimental study not cause cerebral vasoconstriction and hypoperfusion. Further studies are required before NE is routinely implemented in early treatment of haemorrhagic shock.

## Data Availability

The data sets used and analysed during the current study are available from the corresponding author on reasonable request.

## Abbreviations

CBF: Cerebral blood flow
CPP: Cerebral perfusion pressure
CSF: Cerebrospinal fluid
ICP: Intracranial pressure
LP ratio: Lactate to pyruvate ratio
MAP: Mean arterial pressure
NE: Norepinephrine
PbtO_2_: Brain tissue oxygen pressure
